# Predictors of Clinical Success in Patients Selected for Endoscopic Management of Gastrointestinal Transmural Defects

**DOI:** 10.1055/a-2945-3893

**Published:** 2026-09-11

**Authors:** Irene Navarrete-Pérez, Vicente Lorenzo-Zúñiga, Marco Bustamante-Balén, Noelia Alonso-Lazaro, María García Campos, Carla Satorres, Carlos Soutullo-Castiñeiras, Sonia García-García, Lidia Argüello Viudez, Vicente Pons Beltrán

**Affiliations:** 1 Department of Gastroenterology and Endoscopy Unit, Instituto de Investigación Sanitaria La Fe (IIS La Fe) 16273 Hospital Universitari i Politècnic La FeValenciaValencian CommunitySpain; 2 Department of Medicine 253344 Valencia Catholic University Saint Vincent Martyr Faculty of Medicine and Health SciencesValenciaValencian CommunitySpain

**Keywords:** endoscopic management, gastrointestinal defects, leaks, clinical success, prediction model

## Abstract

**Background and Study Aim** We aimed to identify factors associated with clinical success after endoscopic treatment of gastrointestinal transmural defects and to develop an exploratory internally validated prediction model.

**Methods** We retrospectively analyzed consecutive patients treated endoscopically for gastrointestinal transmural defects between January 2014 and December 2024. Technical success was assessed at the procedure level, whereas clinical success and prognostic modeling were analyzed at the patient level to avoid overrepresentation of repeated procedures. Repeated interventions for the same defect were considered part of the same therapeutic episode. Clinical success was defined as complete defect resolution without the need for additional surgical intervention. A ridge-penalized logistic regression model with bootstrap internal validation was developed.

**Results** Ninety-five endoscopic procedures were performed in 83 patients, predominantly for postoperative (79.5%) and upper gastrointestinal (95.2%) defects. Procedure-level technical success was achieved in 89/95 procedures (93.7%). Patient-level clinical success was achieved in 50/83 patients (60.2%). Larger defect size was independently associated with lower probability of clinical success (OR 0.90 per mm increase, 95% CI 0.83–0.99; *p* = 0.026), whereas negative-pressure therapy showed a positive association trend (OR 9.27, 95% CI 0.88–97.81; *p* = 0.064). Model discrimination after bootstrap internal validation was moderate, with reasonable agreement between predicted and observed outcomes.

**Conclusions** Endoscopic management of gastrointestinal transmural defects achieves high technical success but moderate clinical success in real-world practice. An exploratory internally validated prediction model may support risk stratification, although external validation is required before routine clinical use.

## Introduction

Gastrointestinal transmural defects constitute one of the most severe complications in digestive practice. Their incidence has increased in parallel with the rising complexity and volume of surgical and endoscopic procedures, and they remain associated with considerable morbidity and postoperative mortality. 1 2 These defects include acute wall disruptions, postoperative leaks, and fistulous communications, which may vary in chronicity and complexity and often become more challenging when associated with collections or mature tracts. 3

Clinical presentation is frequently subtle or nonspecific, leading to delays in diagnosis. Imaging and endoscopy are therefore essential not only to confirm the presence of a defect but also to characterize its origin, extent, and associated collections to plan appropriate therapy. 2 4

Management requires a multidisciplinary approach based on stabilization, infection control, drainage, nutritional support, and defect closure or exclusion. While surgery continues to be necessary in cases of diffuse peritonitis or uncontrolled sepsis, endoscopic therapy has emerged as a key minimally invasive alternative capable of avoiding reoperation in selected patients. Current endoscopic options include clipping systems, stents, suturing, internal drainage, and negative-pressure therapy, often used in combination according to anatomic and clinical factors. 1 3

Despite increasing use of endoscopic techniques, clinical outcomes remain variable because gastrointestinal transmural defects represent a heterogeneous group of entities with different biological behaviors, chronicity, and healing potential. Furthermore, available evidence is largely based on retrospective series with substantial variability in patient selection and therapeutic strategies. 1 3 5 In this context, identifying clinically relevant predictors of treatment success may help improve risk stratification and individualized decision-making. Therefore, the aim of this study was to evaluate factors associated with clinical success among patients undergoing endoscopic treatment for gastrointestinal transmural defects and to develop an exploratory internally validated prediction model.

## Methods

### Study Design and Population

We conducted a retrospective cohort study at a tertiary referral center in Valencia, Spain, including consecutive patients who underwent endoscopic treatment for gastrointestinal transmural defects between January 2014 and December 2024. The study included postoperative leaks, acute perforations, and fistulous defects treated using endoscopic techniques according to multidisciplinary clinical decision-making. The database comprised 95 procedures performed in 83 patients and was obtained from the institutional Big Data and Biostatistics Unit.

### Cohort Characteristics

Demographic and clinical information was collected at the index endoscopy. Variables included age, sex, ASA physical status, and comorbidities: diabetes mellitus (DM), chronic obstructive pulmonary disease (COPD), left ventricular ejection fraction (LVEF) < 55%, arrhythmia, renal impairment (GFR < 30 mL/min/1.73 m ^2^ ; or 30–90 mL/min/1.73 m ^2^ ), and smoking status. For multivariate modeling, renal impairment was analyzed as a single composite (“renal disease”). Procedure year was recorded and used for descriptive analyses of temporal trends. Etiology was grouped into postsurgical vs non-postsurgical. Procedures were classified as primary endoscopic therapy or rescue therapy following failure of previous interventions.

Anatomic region was recorded and lesions were grouped into upper (esophagus–jejunum) or lower (ileum–rectum) regions. Distance from natural orifices was measured in centimeters: from the incisors for upper-tract lesions, and from the anus for lower-tract lesions. Defect size was measured in millimeters.

Endoscopic methods comprised through-the-scope clips (TTSC; *n* = 14), over-the-scope clips (OTSC; *n* = 5), endoscopic suturing ( *n* = 5), fully covered self-expanding metal stents (FC-SEMS; *n* = 49), and negative-pressure therapy (EsoSponge, *n* = 18; VacStent, *n* = 4). The number of kits used, number of replacements, time to closure (days), complications, stent migration, and stent fixation were also recorded. For prediction modeling, endoscopic treatment was categorized a priori as mechanical closure (TTSC/OTSC/suturing), stent-based containment (FC-SEMS), or negative-pressure therapy (EsoSponge/VacStent).

For exploratory analyses, the study period was additionally categorized into two predefined eras (2014–2021 vs 2022–2024) reflecting progressive incorporation of EVT-based strategies into routine clinical practice at our institution, including increasing use of EsoSponge and VacStent systems at the final period.

### Outcome Definitions

Technical success was assessed at the procedure level and defined as successful deployment of the intended endoscopic device without immediate intraprocedural complications.

Clinical success was assessed at the patient level and defined as complete defect resolution without persistent leakage or need for additional surgical intervention during follow-up, confirmed by imaging, endoscopic reassessment, and clinical evaluation.

Repeated vacuum sponge exchanges, scheduled stent removals, and planned device replacements were considered part of the same therapeutic episode rather than independent clinical outcomes. For patients undergoing multiple procedures, the final outcome was adjudicated at patient level according to overall defect resolution during follow-up.

Because treatment allocation was not randomized and reflected real-world multidisciplinary management, the study was designed as an exploratory prognostic analysis rather than a comparative effectiveness study.

### Statistical Analysis

Statistical analyses were performed using R (R Foundation for Statistical Computing, Vienna, Austria). Descriptive statistics are presented as mean (SD), median (IQR), or percentages. Univariate associations with technical and clinical success were evaluated using Fisher’s exact test for categorical variables and the Wilcoxon rank-sum test for continuous variables; pairwise comparisons of proportions used Holm correction.

For prognostic modeling, candidate predictors were selected a priori according to clinical relevance and included age, sex, ASA class, defect size, anatomic location, distance from natural orifices, and treatment strategy. To reduce overfitting and improve coefficient stability in the context of a relatively limited and heterogeneous cohort, a ridge-penalized logistic regression model was developed.

Model performance was assessed by discrimination (C-index), calibration (calibration slope and intercept), and overall performance (Brier score). The optimism-corrected C-index was 0.70. Internal validation was performed using bootstrap resampling to obtain optimism-corrected estimates. Variables with incomplete retrospective information were analyzed using complete-case methodology.

The prediction model was intended as an exploratory internally validated risk-stratification tool among patients selected for endoscopic treatment rather than a universally applicable model for all gastrointestinal transmural defects.

## Results

### Descriptive Analysis

Between January 2014 and December 2024, 83 patients underwent 95 endoscopic procedures for gastrointestinal transmural defects. Baseline characteristics are summarized in Table 1 . Most patients were male and had significant comorbidity, with nearly 70% classified as ASA III–IV. The majority of defects were postoperative and located in the upper gastrointestinal tract. Among the 83 included patients, 44 (53.0%) underwent index endoscopic treatment during the pre-EVT implementation period (2014–2021), whereas 39 (47.0%) were treated after incorporation of routine EVT-based strategies (2022–2024).

**Table 1:** Patient and defect characteristics ( *n* = 83).

Variable	Value
Age (years)	62.8 ± 11.4 (21–85)
Sex, male	58 (69.9%)
ASA, III–IV	56 (67.5%)
DM	20 (24.1%)
COPD	19 (22.9%)
LVEF <55%	2 (2.4%)
Arrhythmia	17 (20.5%)
Renal impairment (GFR <90 mL/min/1.73 m ^2^ )	33 (39.8%)
Smoking (current/former)	59 (71.1%)
Year of procedure	2014–2021: 44 (53.0%)
	2022–2024: 39 (47.0%)
Etiology	Postsurgical: 66 (79.5%)
	Non-postsurgical: 17 (20.5%)
Anatomical area	Upper GI: 79 (95.2%)
	Lower GI: 4 (4.8%)
Distance from natural orifices, cm	27.7 ± 9.7 (5–75)
Defect size, mm	12.5 ± 14.6 (1–70)

*Abbreviations: ASA, American Society of Anesthesiologists; COPD, chronic obstructive pulmonary disease; DM, diabetes mellitus; GFR, glomerular filtration rate; LVEF, left ventricular ejection fraction.*

The mean defect size was 12.5 mm, although substantial heterogeneity was observed regarding anatomic location, chronicity, and therapeutic complexity. Endoscopic treatment was performed as primary therapy in most cases, whereas rescue procedures following previous treatment failure accounted for a smaller but clinically complex subgroup ( Table 2 ).

**Table 2:** Procedural characteristics and outcomes ( *n* = 95).

Variable	Value
Endoscopic technique	FC-SEMS: 49 (51.6%)
	TTSC: 14 (14.7%)
	OTSC: 5 (5.3%)
	Endoscopic suturing: 5 (5.3%)
	EsoSponge: 18 (18.9%)
	VacStent: 4 (4.2%)
Indication	Primary: 78 (82.1%)
	Rescue: 17 (17.9%)
Technical success	89 (93.7%)
Number of kits	2.0 ± 1.7 (1–12)
Number of replacements	0.7 ± 1.6 (0–11)
Time to closure, days	30.1 ± 38.8 (0–238)
Complications	6 (6.3%)
Stent migration	15/49 (30.6%)
Stent fixation	32/49 (65.3%)

*Abbreviations: FC-SEMS, fully covered self-expanding metal stent; OTSC, over-the-scope clip; TTSC, through-the-scope clip.*

### Technical Success

Technical success was achieved in 89/95 procedures (93.7%). Technical success rates differed across endoscopic techniques (TTSC, *n* = 14; OTSC, *n* = 5; endoscopic suturing, *n* = 5; FC-SEMS, *n* = 49; EsoSponge, *n* = 18; VacStent, *n* = 4), although interpretation is limited by the relatively small size of several treatment subgroups ( Fig. 1 ). Fully covered self-expanding metal stents and negative-pressure therapies demonstrated consistently high deployment success rates, whereas mechanical closure techniques showed greater variability.

**Fig. 1:**
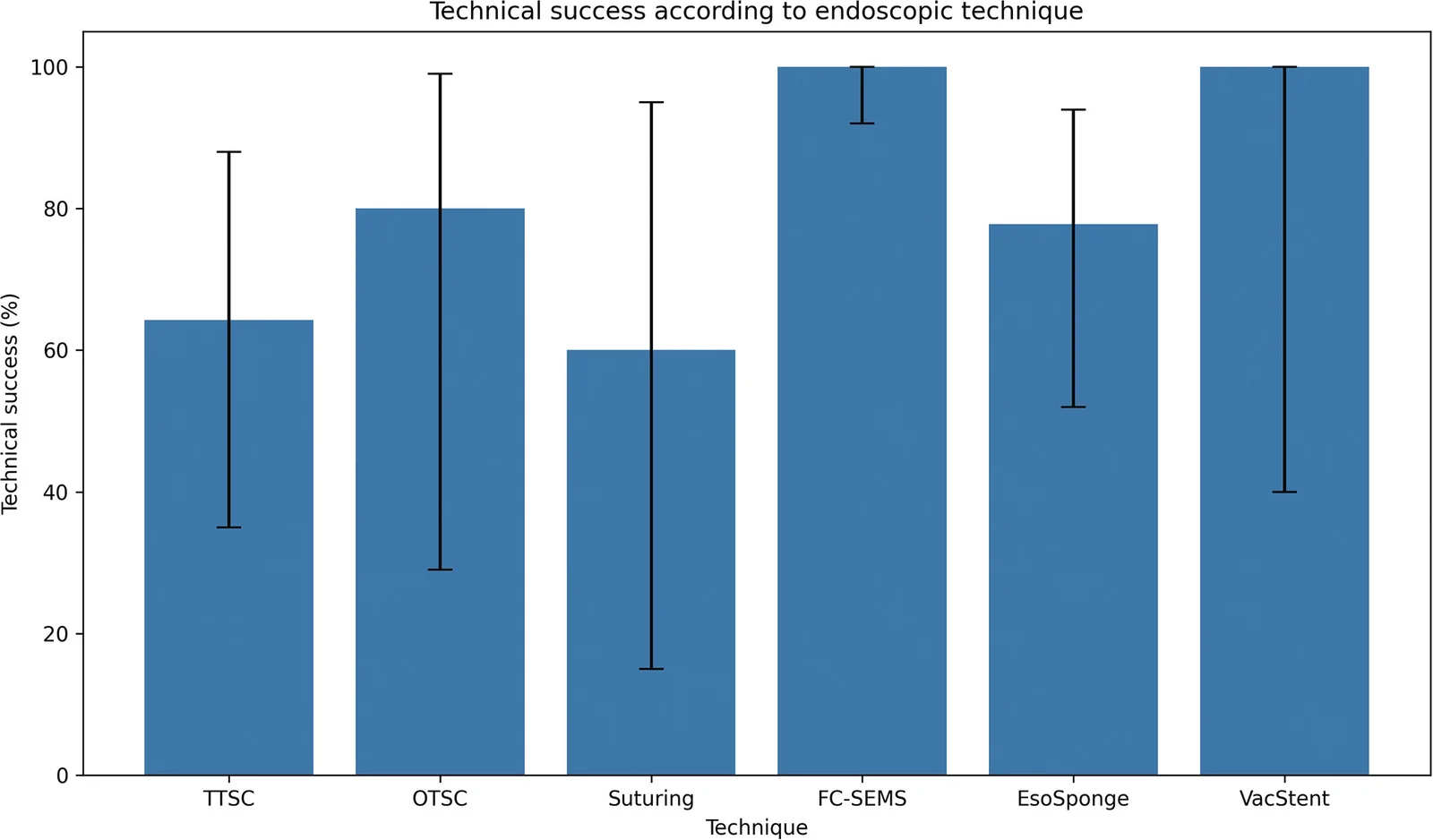
Technical success according to endoscopic technique. Bars represent technical success rates with 95% confidence intervals. Negative-pressure and stent-based therapies demonstrated consistently high deployment success, whereas mechanical closure techniques showed greater variability.

No significant temporal association between procedural year and technical success was observed ( Fig. 2 ). However, temporal analyses should be considered descriptive because yearly subgroup sizes were limited and therapeutic strategies evolved substantially during the study period.

**Fig. 2:**
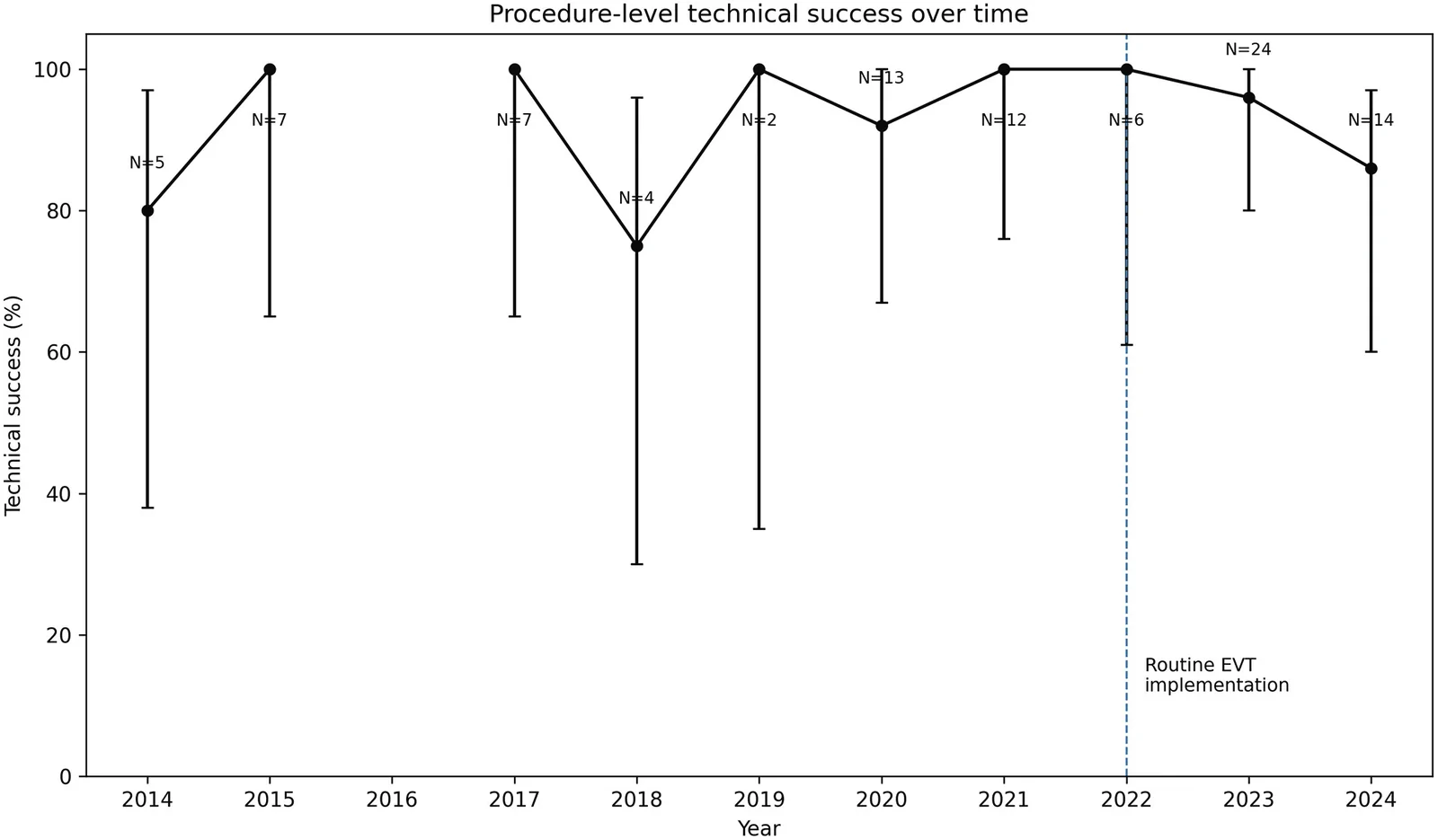
Technical success over time. Annual procedure-level technical success rates are shown with 95% confidence intervals. No procedures were performed in 2016.

### Clinical Success

At patient level, complete clinical success was achieved in 50/83 patients (60.2%). Patient-level clinical success according to endoscopic technique is shown in Fig. 3 .

**Fig. 3:**
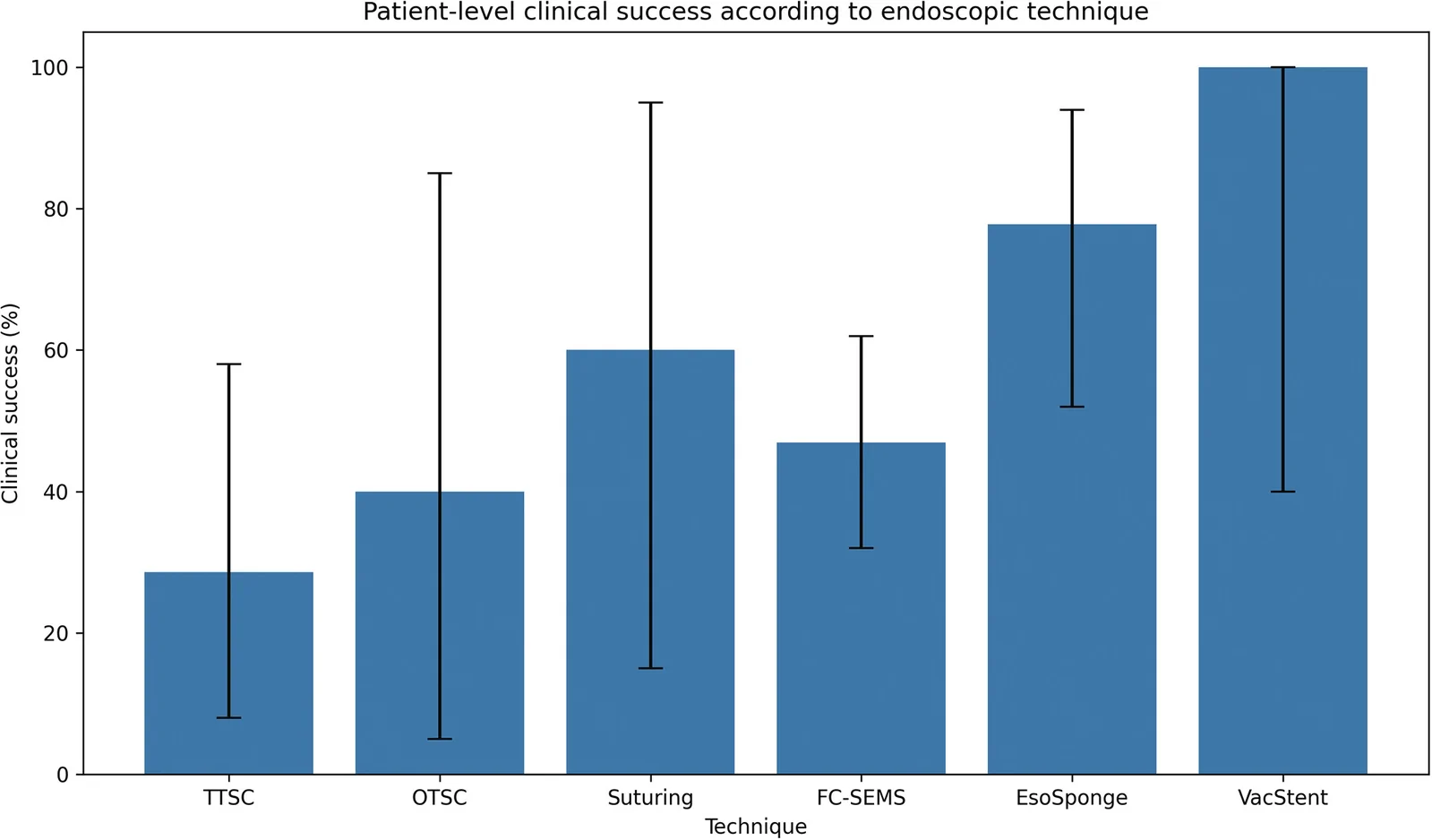
Patient-level clinical success according to endoscopic technique. Bars represent clinical success proportions with 95% confidence intervals. Interpretation of subgroup comparisons should remain cautious because of limited sample sizes and cohort heterogeneity.

Clinical outcomes varied substantially according to defect complexity and therapeutic strategy. Negative-pressure therapies appeared associated with higher healing rates in selected patients with complex postoperative defects, particularly when active drainage and infection control were clinically relevant. In contrast, mechanical closure techniques were more frequently used in smaller or technically accessible defects but demonstrated greater variability in long-term healing.

Larger defect size and higher ASA class were associated with lower probability of clinical success, suggesting that both local defect severity and overall physiologic vulnerability influence healing outcomes. Acute perforations appeared to show more favorable evolution than chronic fistulous defects, although the study was not powered for formal subgroup comparisons.

Temporal trends in clinical success are presented in Fig. 4 . Clinical success appeared numerically higher in the later years of the study, particularly after 2022. Importantly, this temporal trend likely reflects multiple concurrent factors, including increasing institutional experience, refinement of multidisciplinary management pathways, earlier referral patterns, and progressive implementation of drainage-based endoscopic strategies such as EVT, including EsoSponge and VacStent systems. Therefore, temporal comparisons should be interpreted cautiously and not considered evidence of isolated treatment effects or learning-curve superiority.

**Fig. 4:**
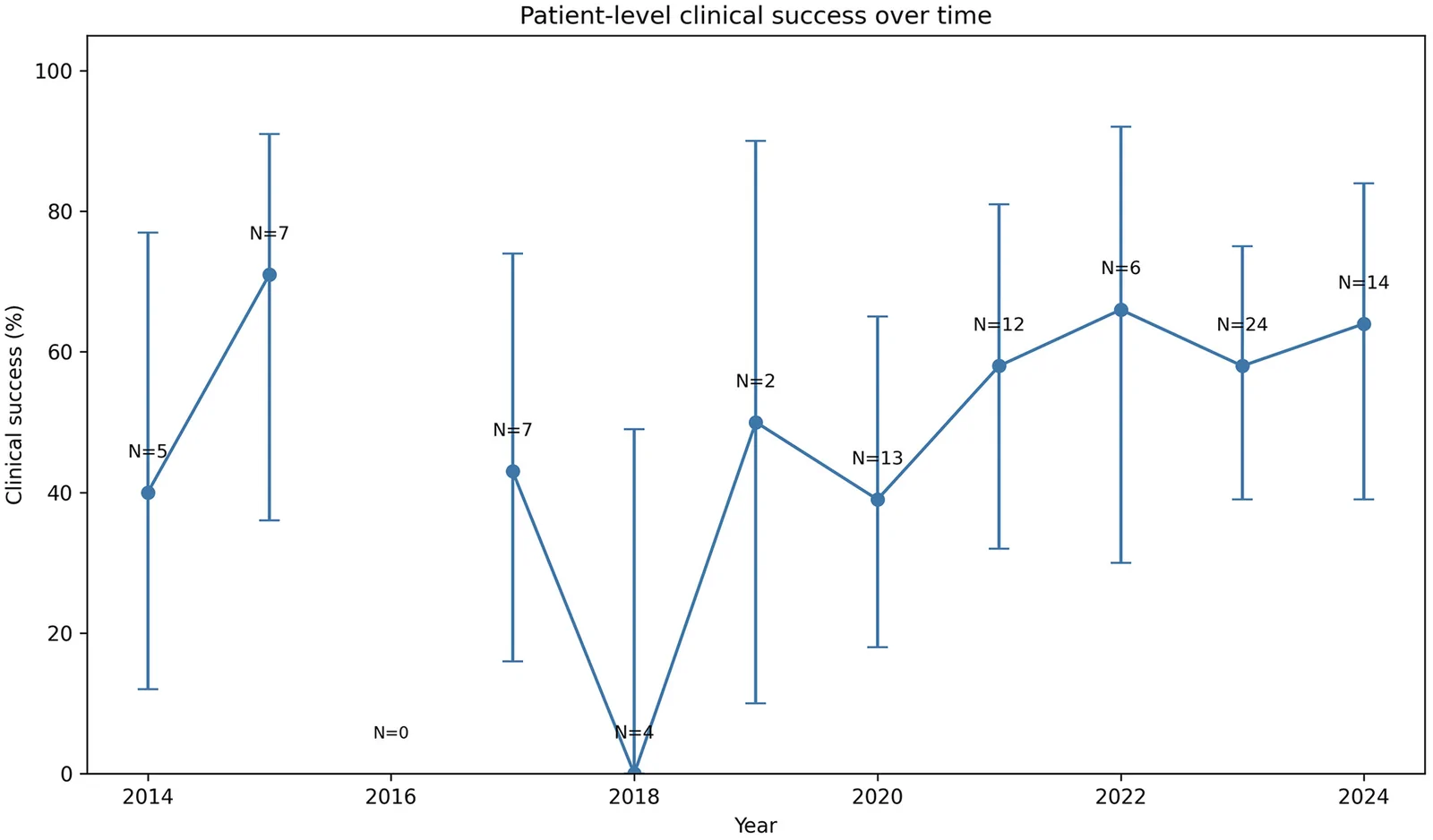
Patient-level clinical success over time. Annual clinical success rates are shown with 95% confidence intervals. Interpretation of temporal trends should remain cautious because yearly subgroup sizes were limited and therapeutic strategies evolved substantially during the study period.

### Exploratory Prognostic Model

An exploratory ridge-penalized logistic regression model was developed to identify variables associated with clinical success at patient level. Candidate predictors were selected a priori according to clinical relevance and included demographic, anatomic, and therapeutic variables.

Each 1-mm increase in defect size was associated with approximately 10% lower odds of clinical success (OR 0.90 per-mm increase, 95% CI 0.83–0.99; *p* = 0.026). Negative-pressure therapy demonstrated a strong positive association trend (OR 9.27, 95% CI 0.88–97.81; *p* = 0.064), although confidence intervals remained wide because of the limited sample size and subgroup heterogeneity. Other variables, including ASA class, anatomic location, and post-2022 treatment era, were not independently associated with outcome after adjustment.

Model discrimination after bootstrap internal validation was moderate, and calibration analyses suggested reasonable agreement between predicted and observed outcomes. Nevertheless, because of the relatively limited sample size, heterogeneous cohort composition, and retrospective design, the model should be interpreted as exploratory and hypothesis-generating rather than definitive or treatment-directive. Full model coefficients are presented in **Supplementary Table S1** .

### Exploratory Nomogram

Based on the final exploratory penalized model, a nomogram was constructed to estimate individualized probability of clinical success among patients selected for endoscopic treatment ( Fig. 5 ). The nomogram integrates clinically accessible variables including physiological status, defect size, anatomic characteristics, and therapeutic strategy.

**Fig. 5:**
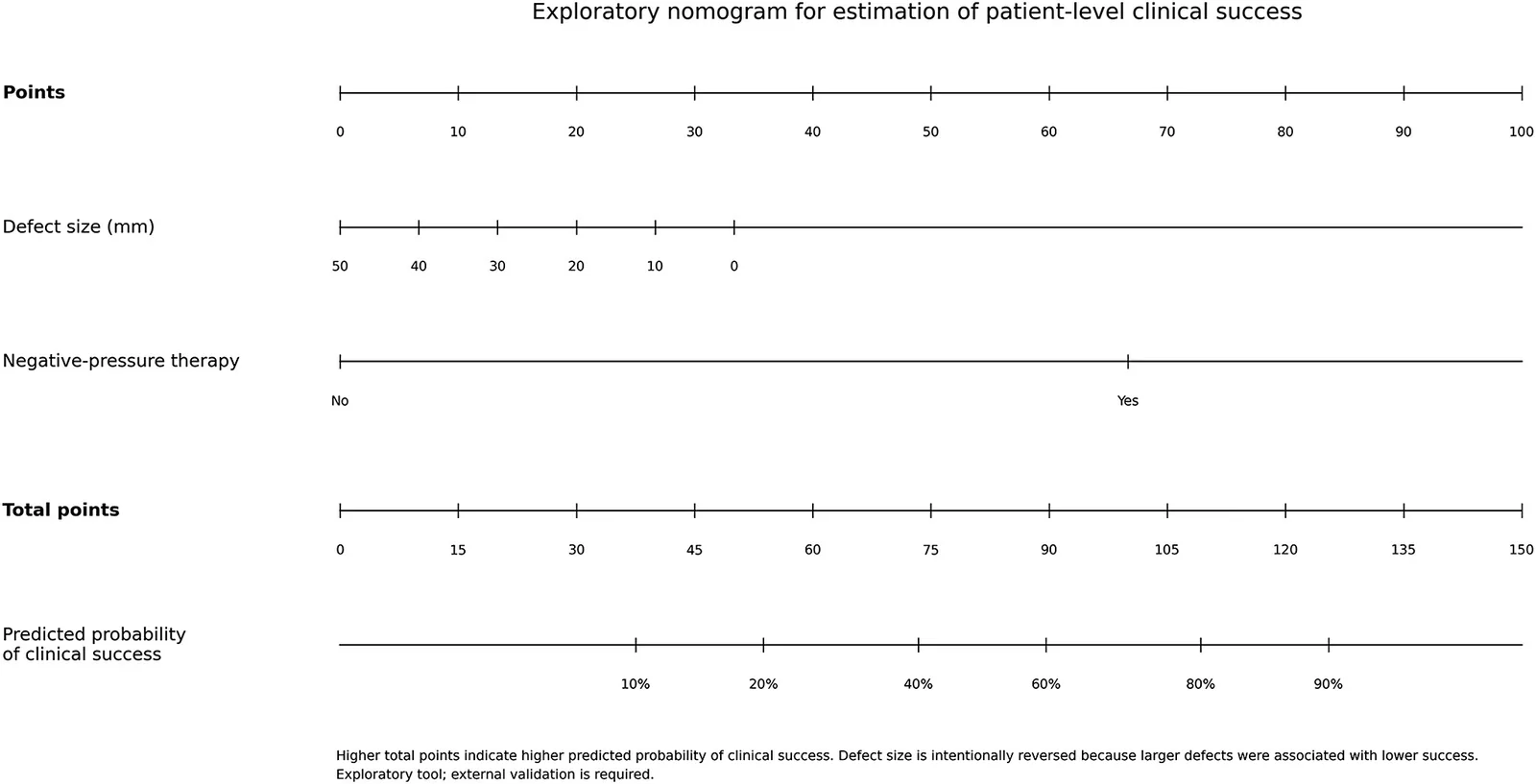
Exploratory internally validated nomogram derived from the penalized logistic regression model for estimation of patient-level clinical success among patients selected for endoscopic treatment.

The model is intended as an exploratory internally validated risk-stratification tool rather than a comparative effectiveness instrument or definitive clinical decision-making algorithm. Model discrimination and calibration are shown in **Supplementary Figs. 1 and 2** .

## Discussion

This retrospective single-center study evaluated factors associated with clinical success among patients undergoing endoscopic treatment for gastrointestinal transmural defects in routine clinical practice. The principal findings were: (i) endoscopic treatment achieved very high technical success but more moderate clinical success; (ii) defect size, physiological status, and therapeutic strategy appeared associated with healing probability; and (iii) the proposed internally validated model may provide exploratory risk stratification among patients selected for endoscopic management.

A major challenge in this field is the substantial heterogeneity of gastrointestinal transmural defects. Acute iatrogenic perforations, postoperative leaks, and chronic fistulas represent biologically distinct entities with important differences in contamination, fibrosis, inflammatory burden, chronicity, and intrinsic healing potential. 1 Consequently, therapeutic response is highly variable and difficult to standardize. 2 The present cohort reflects this real-world complexity, which enhances clinical applicability but simultaneously limits the precision of disease-specific predictive modeling.

Negative-pressure therapies appeared associated with more favorable healing trends in selected patients, particularly in complex postoperative defects requiring continuous drainage and local infection control. These findings are consistent with the growing literature supporting endoscopic vacuum therapy in upper gastrointestinal leaks and anastomotic complications. Nevertheless, because treatment allocation was not randomized and reflected multidisciplinary clinical judgment, the present study should not be interpreted as evidence of comparative superiority between therapeutic modalities. Indeed, confounding by indication likely influenced treatment selection, especially in more severe or chronic defects.

The proposed nomogram should therefore be interpreted cautiously as an exploratory internally validated framework for risk estimation among patients already selected for endoscopic treatment. Its primary value lies in supporting clinical stratification and hypothesis generation rather than directing treatment selection. Importantly, the model was not designed to determine candidacy for endoscopic treatment itself, but rather to estimate probability of success among patients already selected for endoscopic management within multidisciplinary clinical practice. External validation in larger multicenter cohorts with more homogeneous subgroup representation will be necessary before considering broader clinical implementation.

Our findings align with the existing literature on endoscopic management of perforations, leaks, and fistulas. Prior studies consistently report technical success rates above 80% for modalities including clips, stents, suturing, and EVT, whereas clinical success varies from roughly 50% to 90% across series. 1 2 5 6 7 8 This variability reflects the heterogeneous nature of transmural defects; in particular, chronic fistula tracts have markedly lower healing rates. 1 6 7

The moderate overall clinical success in our study falls within this reported range and underscores the need for continued improvements in therapy. Importantly, accumulating evidence favors *negative-pressure therapies* over traditional stents for many defects. EVT has demonstrated higher closure rates, shorter treatment durations, and lower morbidity compared to fully covered metal stents in both comparative studies and meta-analyses. 9 10 11 12 These advantages are attributed to EVT’s ability to continuously drain fluid and control infection, thereby promoting granulation and healing, 6 13 whereas stent therapy alone is limited by issues such as migration, tissue ingrowth, and inadequate drainage. 9 Reflecting this paradigm shift, novel hybrid devices like the *VacStent* (a fully covered stent with integrated vacuum suction) have shown promising early results with around 77% clinical success with the VacStent in esophageal leaks. 8 Taken together, both our data and the broader literature underscore that drainage-based endoscopic strategies have become a cornerstone of managing GI transmural defects, offering superior outcomes in suitable patients.

Several limitations should be acknowledged. First, this was a retrospective single-center study with a relatively limited sample size and substantial clinical heterogeneity. Second, the cohort included biologically distinct entities including acute perforations, postoperative leaks, and chronic fistulas, which may exhibit markedly different healing behavior and therapeutic response. Third, treatment allocation was not randomized and therefore susceptible to treatment-selection bias and confounding by indication. Fourth, some timing-related variables, including precise delay from diagnosis to intervention and chronicity classification, were incompletely available retrospectively and could not be systematically incorporated into the final model. Fifth, although clinical success was redefined and analyzed at patient level to avoid overrepresentation of repeated therapeutic sessions, residual bias related to therapeutic complexity cannot be completely excluded. Additionally, some patients underwent sequential or combined endoscopic therapies during the same therapeutic episode, further limiting causal interpretation of treatment-specific associations. Accordingly, the proposed model should be interpreted as exploratory and hypothesis-generating and requires external validation before routine clinical application.

In conclusion, endoscopic management of gastrointestinal transmural defects achieves high technical success in real-world clinical practice, although definitive healing remains more variable and strongly dependent on patient and defect characteristics. Smaller defects, lower physiologic risk, and drainage-based therapies appeared associated with more favorable outcomes. The proposed exploratory internally validated prediction model may help support individualized risk assessment among patients selected for endoscopic therapy; however, external validation is required before routine clinical application.

## Supplementary Material

Ergänzendes Material Supplementary Material
