# Supplementary material for: Predictors of Clinical Success in Patients Selected for Endoscopic Management of Gastrointestinal Transmural Defects
Source: Endosc Int Open. 2026 Sep 11;14:a29453893. doi: 10.1055/a-2945-3893 (PMC13641219; doi:10.1055/a-2945-3893)
Supplement: Supplementary file 1 [file 10-1055-a-2945-3893_29502565.pdf]

Supplementary Material

Predictors of Clinical Success in Patients Selected for Endoscopic Management of Gastrointestinal Transmural Defects

Irene Navarrete-Pérez, Vicente Lorenzo-Zúñiga, Marco Bustamante-Balén, Noelia Alonso, María García-Campos, Carla Satorres, Carlos Soutullo-Castiñeiras, Sonia García-García, Lidia Argüello, Vicente Pons-Beltrán.

Supplementary Table S1

Exploratory patient-level logistic regression model for clinical success (n=83 patients).

**Predictor**	**OR**	**95% CI**	**p value**
Defect size (per 1 mm increase)	0.90	0.83–0.99	0.026
Negative-pressure therapy	9.27	0.88–97.81	0.064
ASA class	1.00	0.46–2.17	0.99
Upper GI location	0.84	0.07–10.79	0.90
Post-2022 EVT era	1.85	0.50–6.95	0.36

Odds ratios are derived from the revised patient-level exploratory logistic regression model. Interpretation should remain cautious because of the limited sample size and cohort heterogeneity.

Supplementary Figure S1

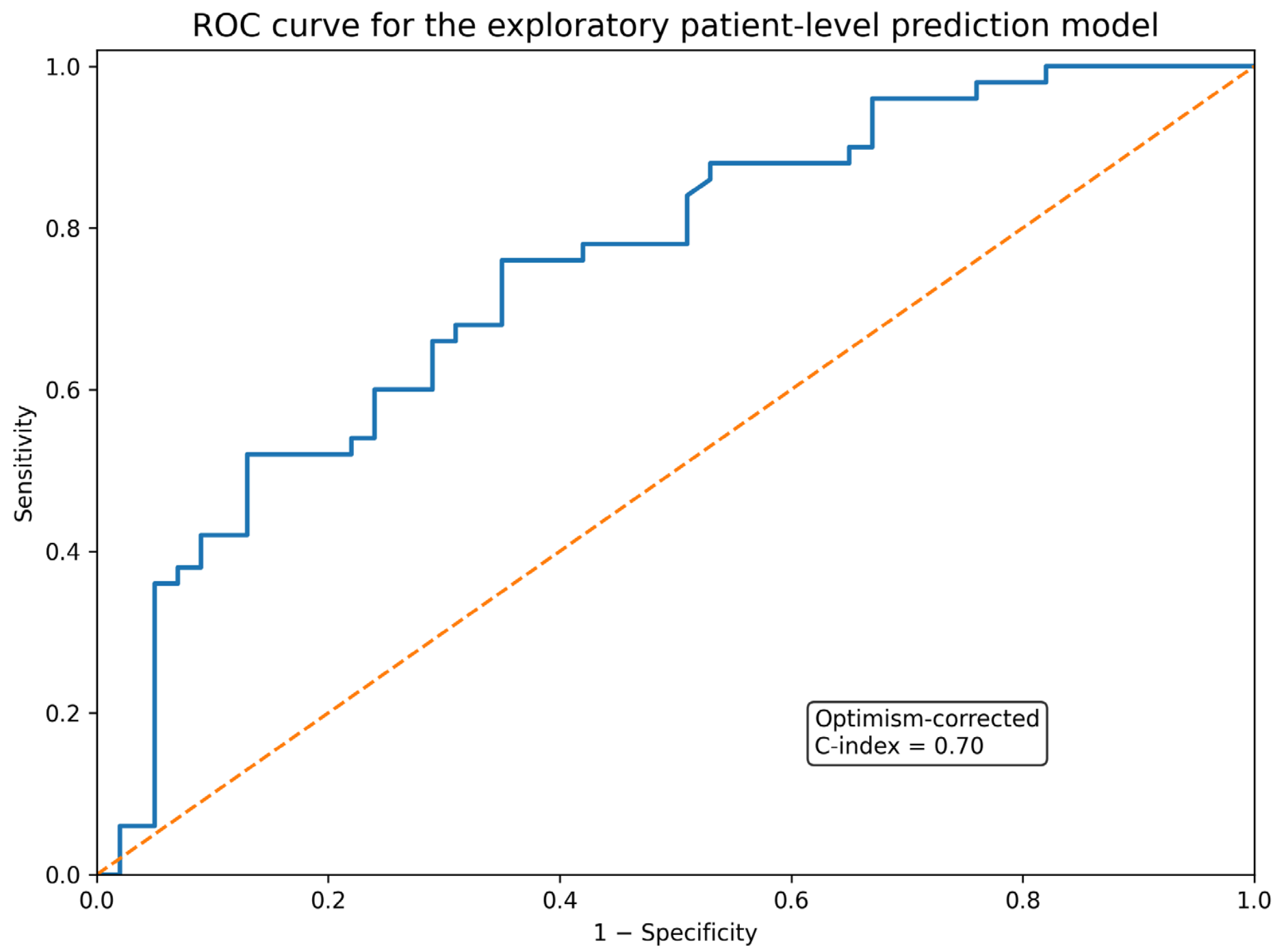

Supplementary Figure S2

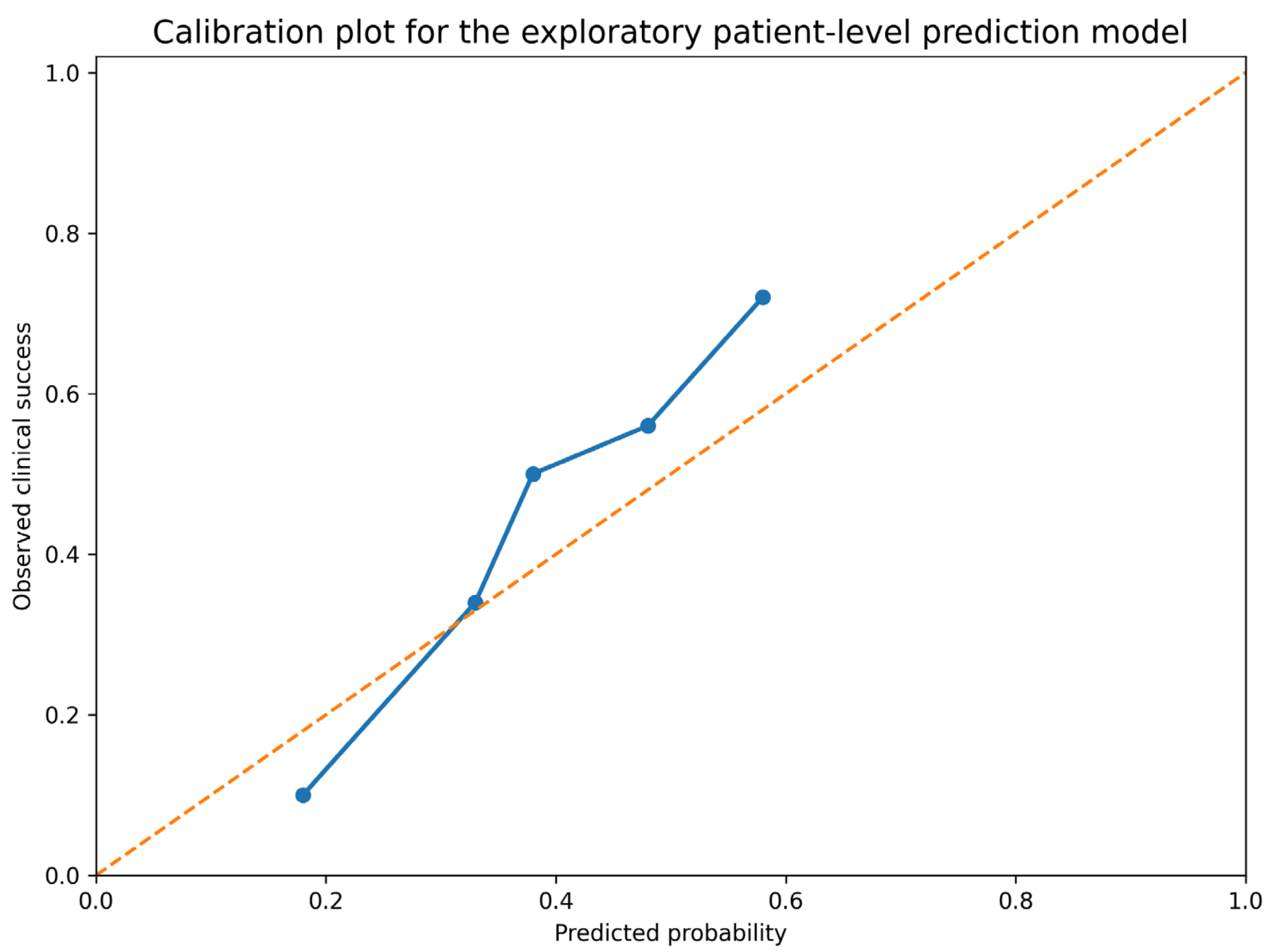

Endosc Int Open | DOI 10.1055/a-2945-3893 | © The Author(s).
